# Matataki: an ultrafast mRNA quantification method for large-scale reanalysis of RNA-Seq data

**DOI:** 10.1186/s12859-018-2279-y

**Published:** 2018-07-16

**Authors:** Yasunobu Okamura, Kengo Kinoshita

**Affiliations:** 1 Graduate School of Information Sciences, Tohoku University, Sendai, Miyagi Japan; 2 Mitsubishi Space Software Co., Ltd, Amagasaki, Hyogo Japan; 3 Tohoku Medical Megabank Organization, Sendai, Miyagi Japan; 4 Institute of Development, Tohoku University, Sendai, Miyagi Japan

**Keywords:** RNA-Seq, Mapping, Gene expression

## Abstract

**Background:**

Data generated by RNA sequencing (RNA-Seq) is now accumulating in vast amounts in public repositories, especially for human and mouse genomes. Reanalyzing these data has emerged as a promising approach to identify gene modules or pathways. Although meta-analyses of gene expression data are frequently performed using microarray data, meta-analyses using RNA-Seq data are still rare. This lag is partly due to the limitations in reanalyzing RNA-Seq data, which requires extensive computational resources. Moreover, it is nearly impossible to calculate the gene expression levels of all samples in a public repository using currently available methods. Here, we propose a novel method, Matataki, for rapidly estimating gene expression levels from RNA-Seq data.

**Results:**

The proposed method uses k-mers that are unique to each gene for the mapping of fragments to genes. Since aligning fragments to reference sequences requires high computational costs, our method could reduce the calculation cost by focusing on k-mers that are unique to each gene and by skipping uninformative regions. Indeed, Matataki outperformed conventional methods with regards to speed while demonstrating sufficient accuracy.

**Conclusions:**

The development of Matataki can overcome current limitations in reanalyzing RNA-Seq data toward improving the potential for discovering genes and pathways associated with disease at reduced computational cost. Thus, the main bottleneck of RNA-Seq analyses has shifted to achieving the decompression of sequenced data. The implementation of Matataki is available at https://github.com/informationsea/Matataki.

**Electronic supplementary material:**

The online version of this article (10.1186/s12859-018-2279-y) contains supplementary material, which is available to authorized users.

## Background

The number of published studies on RNA sequencing (RNA-Seq) data is rapidly increasing owing to improvements in RNA-Seq measurement technology. Thus, meta-analyses of publicly available data have become a new promising approach to obtain novel insights into biological systems. However, merging quantified expression data provided by authors is generally difficult because of the use of different reference sequences, ID systems, and quantification methods among individual studies. These variations make it impossible to distinguish true biological differences from calculation protocol biases when comparing gene expression profiles quantified using different methods. Therefore, quantification using raw sequences for all data is an important step for RNA-Seq meta-analyses.

Many quantification methods for RNA-Seq data have been proposed to date, including the most common pipeline method using TopHat2 [1, 2] and cufflinks [3]. This method aligns sequenced reads to a reference genome, counts the number of fragments mapped onto gene regions, and estimates gene expression as transcript levels. Importantly, this method can be applied to species without a reference transcript and can predict transcript candidates. Some other methods such as RSEM [4] and eXpress [5] map sequences to the transcript reference; since they require only reference transcript sequences, they can be applied to species without a reference genome. A de novo transcript assembler or an expressed sequence tag database can be used as reference transcript sequences in place of curated reference transcript databases. Both RSEM and eXpress employ bowtie [6] to map a read sequence to a transcript, and some read sequences are mapped to multiple transcripts due to splicing variants. RSEM and eXpress use the Expectation-Maximization (EM) algorithm to resolve the problem of assigning multi-mapped reads to transcripts for quantifying the expression level of transcripts.

Despite their advantages for quantification, these alignment-based methods require extensive computational resources. When quantifying the expression levels of an RNA-Seq sample, alignment is an optional step because the position of a read is not essential for quantification. Thus, several methods have also been proposed to reduce the calculation cost for large RNA-Seq analyses and avoid the mapping step by focusing on the k-mers in transcripts. For example, Sailfish [7] uses all k-mers that appear in the reference transcript, creates a transcript table containing the k-mers, counts the number of occurrences of each k-mer in the RNA-Seq data, and finally estimates the most probable expression level of each transcript from the counts using the EM algorithm. RNA-Skim [8] uses a similar but more efficient approach by introducing *sig-mers* that appear only once in a subset of reference transcripts, counts the number of occurrences of the *sig-mers* while processing the RNA-Seq data, and then estimates the most probable expression levels using the EM algorithm. Kallisto [9] also uses k-mers, and further reduces the calculation cost by skipping fragments when searching an index. When a k-mer appears, the next k-mer is limited to one or a few patterns. If the next k-mer is limited to one pattern, hashing the k-mer is not required to determine the source isoform. Kallisto then skips these non-informative k-mers, resulting in a faster estimation process.

The speed of quantification is a critical step in developing a method to process thousands of publicly available RNA-Seq reads. Although these alignment-free methods such as Sailfish, Kallisto, and RNA-Skim are much faster than the alignment-based methods, the recent accumulation of large-scale sequence data requires development of an even faster method for data management and reanalysis. In addition, all of these alignment-free methods rely on transcript-level quantification, although gene-level expression data contain sufficient information for most analyses. Moreover, several RNA-Seq studies [10–13] do not include isoform-specific expression data; even if isoform-specific expression is relevant, these analyses typically only focus on a few splicing changes [14, 15]. For example, Wu et al. [14] performed gene-level quantification for all genes initially, followed by isoform-level quantification. Therefore, gene-level expression data are useful in many cases. In particular, large-scale reanalysis of human and mouse RNA-Seq data such as in gene co-expression analysis [16] or comparison of similar expression profiles does not require precise expression data at the transcript level. For example, the growing the number of expression profiles provides a better quality gene co-expression dataset [17]. In this case, simple gene-level quantification is sufficiently accurate, which can then be improved by transcript-level estimation [18].

To further enhance large-scale meta-analyses of RNA-Seq data, we here propose a new quantification algorithm called Matataki. Similar to Kallisto, our method uses k-mers that appear only once in a gene and quantifies expression from the number of *unique* k-mers. However, our method has an additional advantage of reducing computational costs with the integration of two novel approaches. First, Matataki quantifies expression directly without implementation of the EM algorithm by focusing on the gene level. Second, our method checks fragments of reads at fixed skips even if the k-mer was not indexed. Because k-mers unique to a gene are usually found continuously, hashing all fragments of a read does not improve performance. Thus, Matataki provides a novel approach for ultra-fast RNA-Seq quantification based on unique k-mers to each gene. More specifically, our method searches for all k-mers that appear only once in a gene among a set of transcripts in only two steps: an index building step and a quantifying expression step. Here, we describe the proposed method and its implementation, and compare the performance against available methods using reference sequence and simulation datasets as test data.

## Methods

### Index building step

To achieve a fast mapping process, Matataki has to search for all k-mers that are unique to each gene. When multiple transcripts are available for a gene, the selected k-mers should include all isoforms of the gene to avoid any effects of the differential expression of isoforms.

First, Matataki searches all unique k-mers to each gene in consideration of all k-mers in all transcript sequences. To judge the uniqueness of the k-mers, Matataki stores the k-mers in a hash table. Except in cases of a strand-specific read, all reverse complements of the k-mers are also considered. Second, Matataki checks whether all of the isoforms of a gene have a k-mer. Because Matataki quantifies expression at the gene level, differences in isoform-specific expression will be ignored. In other words, Matataki builds an index of k-mers that are unique to a gene and are found in all isoforms of the gene. Finally, Matataki counts the number of indexed k-mers for each gene, which will be used to determine the fragment per kilobase of million (FPKM) and transcript per million (TPM) values that are used in the quantification step. The pseudocode is shown in Additional file 1: Method S1. This building step is required only once for each species before using Matataki.

### Quantification step

The quantification step can be divided into two sub-steps: counting the k-mers, and calculating FPKM and TPM values from the read counts.

First, Matataki searches the indexed k-mers in a short read obtained through a next-generation sequencing experiment. When a read has k-mers associated with a gene, it is assigned to that gene. Matataki then counts the number of reads assigned to each gene. When a read has k-mers from two or more genes, the read will be excluded from further analyses.

In the first step, the identified k-mers tend to be found sequentially; thus, we considered that searching all fragments of reads in a step-by-step manner is not required. Therefore, Matataki creates k-mers in step-size (*S*) base intervals instead of creating all possible k-mers from a sequenced read so as to reduce the number of k-mer searches, and ultimately the computational time and cost. We also introduced the “accept-count” parameter *M*, which is the minimum number of matched k-mers required to select a gene, to avoid the noise caused by fragments of a read sequence that matched to an indexed k-mer by chance. A read without an *M* times match to a gene is neglected because it is considered to have potentially matched by chance. Since some reads might have a sequencing error, mutation, or insertion/deletion, a fragment of a read might incorrectly match to an indexed k-mer. Usually, these incorrect matches are not found consecutively in a read; thus, the accept-count parameter *M* helps to avoid this type of incorrect match. When processing a pair-end sequenced file, each read is processed separately. The pseudocode is shown in Additional file 1: Methods S2.

In the next step, Matataki calculates FPKM and TPM from gene-specific read counts using the following formulas:

1 $$ {F}_i=\frac{C_i/{K}_i}{\sum_j{C}_j}{10}^9 $$

2 $$ {T}_i=\frac{C_i/{K}_i}{\sum_j\left({C}_j/{K}_j\right)}{10}^6 $$

where *F*_*i*_ is FPKM, *T*_*i*_ is TPM, *C*_*i*_ is the count of gene-specific reads, and *K*_*i*_ is the number of indexed k-mers in a gene. Because Matataki uses only gene-specific k-mers, the EM or another algorithm is not needed to calculate the expression levels.

### Implementation

We implemented Matataki with C++ 03, autotools, and KyotoCabinet [19]. To reduce memory usage and increase speed, a hash table format was optimized for the RNA/DNA k-mers. The first 4 K bytes contain the header of an index, including the number of entries, size of the hash table, and *k*, and the k-mers and corresponding gene indexes are written after each header. Each entry has two subsections: a gene index and k-mers. A k-mer is compressed as a 2-bit representation of nucleic acids to reduce memory usage and hash value calculation time. Because each k-mer has a fixed length in one index, the entries do not contain length data. The hash function is also important for enabling a quick search of items in the table. We used the fast and widely accepted hash function MurMurHash3 for the hash table. Since building an index requires abundant resource, we distributed the pre-calculated index for publicly available human and mouse sequences.

The source code, pre-built binaries, and pre-calculated index of human and mouse data are available at Github (https://github.com/informationsea/Matataki) and Additional file 2.

### Comparison with other software products

We compared the performance of Matataki with that of the currently available quantification methods bowtie 1.1.2 [6]/eXpress 1.5.1 [5], RSEM 1.2.22 [4], Sailfish 0.10.0 [7], and Kallisto 0.44.0 [9]. These comparisons were carried out using the default parameters of each software. We used binary-distributed files for bowtie/eXpress. Matataki, Sailfish, Kallisto, and RSEM were compiled with GCC 5.2.0. For this study, all running times and memory usages were measured in cluster machines. Each cluster node had two Intel® Xeon® CPU Silver 4116 2.10 GHz and 96 GB RAM.

### Test dataset

We used RefSeq and gene2refseq [20] to create a reference database, which were downloaded on June 26, 2015 from the Human Genome Center, a mirror site of the National Center for Biotechnology Information. In the human RefSeq, 25,894 genes and 55,100 transcripts were available at the time of download. We also used GENCODE version 28 to create a reference database [21].

To examine the quantification quality, we used ERR188125. This run is a part of ERS185259, “RNA-sequencing of 465 lymphoblastoid cell lines from the 1000 Genomes.” The length of reads in ERR188125 was 75, and the number of reads was 28,810,860.

We also compared quantification quality using simulation data. To create the simulation data, we used the rsem-simulate-reads included in RSEM. The simulation models were created by quantifying ERR188074, ERR188125, ERR188171, and ERR188362 with RSEM.

## Results & Discussion

### Statistics of indexed k-mers

#### Number of genes with indexed k-mers

We first checked the number of genes with indexed k-mers in human, mouse, and *Arabidopsis* genomes when the parameter *k* in the considered k-mer was varied from 10 to 100. To effectively compare the results for different species, the numbers were converted to the ratio of genes (i.e., the gene coverage), which are shown in Additional file 1: Figure S1A. For *k* = 10, only a few human genes had unique k-mers in all species, while for *k* = 14, 96.8% of the human genes in RefSeq had indexed k-mers. The coverage of indexed genes reached a maximum at *k* = 34. However, *k* values that were too large resulted in lower gene coverage because some genes had only small transcripts.

Similarly, we evaluated the nucleotide coverages of indexed k-mers, ratio of the number of total indexed position for each transcript, and total length of the transcripts (see Additional file 1: Figure S1B). For the human data, *k* = 14 did not allow for sufficient coverage of sequences with indexed k-mer regions, and the nucleotide coverage almost reached its maximum at *k* = 18. This observation suggested that *k* should be larger than 18 to cover a sufficient number gene-specific gene regions. Similar trends were observed in the mouse and *Arabidopsis* datasets. Because the average length of genes in *Arabidopsis* is smaller than that in human and mouse genes, both gene and nucleotide coverage for *Arabidopsis* at *k* = 10 and 12 were better than those for the other species.

#### Distribution of indexed k-mers in human transcript sequences

To check the distribution of unique k-mers in each gene, we calculated the nucleotide coverage for each human gene at *k* = 32 (see Additional file 1: Figure S2A). As a result, most human genes (86.4%) had a coverage rate higher than 50, and 61% of the human genes had coverage rates higher than 90%, indicating the existence of successive unique k-mers. As this pattern is reminiscent of islands in the sea, we call such a continuous region of nucleotides made from a successive index of k-mers a “cover island”.

To clarify the nature of the cover islands, we checked the number of cover islands and their lengths for each gene (see Additional file 1: Figure S2B, S2C). As a result, 60% of the genes had only one or two cover islands, and the median length of second longest cover island for each gene (327) was much smaller than that of the longest cover island (1262). We determined the existence of successive continuous nucleotides of unique k-mers, designated as “cover islands”, and found that most genes have a main cover island and several small satellite cover islands. Because the lengths of the longest cover islands for each gene were sufficiently longer than the *k* and the step size *S* used in this study, they did not interfere with the quantification accuracy when introducing the step size *S*. It may be noteworthy that all unique k-mers should be listed in the index to implement the idea of step size, indicating that fast heuristic methods such as bloom filter [22] cannot be applied to build the index, as such methods could miss some hits of unique k-mers. Therefore, although introduction of the step size parameter will require a longer time to construct the indexes, for large-scale meta-analyses, the speed of quantification is more important than the speed of building the index. Importantly, our method depends on the quality and completeness of the transcript database. For this assessment, we used RefSeq instead of GENCODE, because GENCODE has less reliable transcripts that are not our target [23].

### Comparison of quantification quality using simulation data

**Fig. 1:**
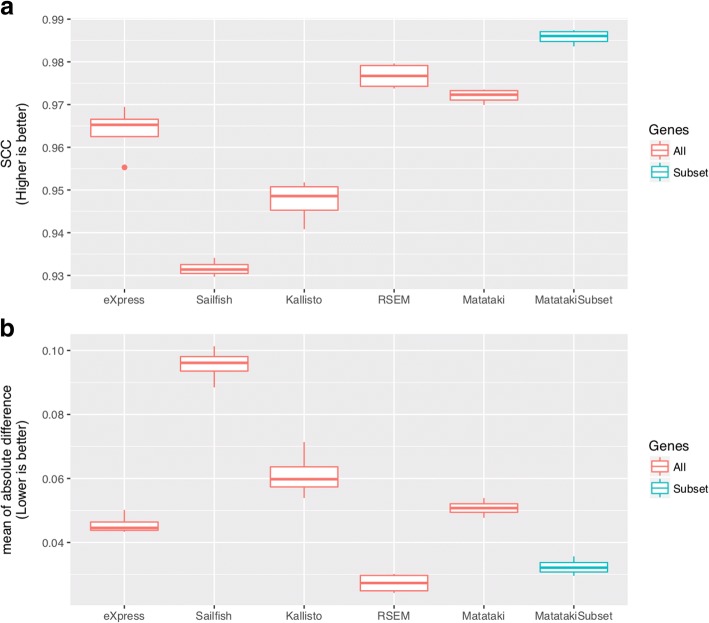
Summary of the results using simulation data. **a** Spearman correlation coefficient with the expected expression and estimated expression values using each method. “Matataki” indicates the results of the proposed method, and “MatatakiSubset” indicates the results of the proposed method without uncovered genes. To compare the gene-level expression profile and transcript-level expression profile, the sum of TPM by each gene was calculated. **b** Means of absolute difference from the expected expression levels

We also compared TPM among eXpress, RSEM, Sailfish, Kallisto, and our method using simulation data. In this comparison, we used *k* = 32, *S* = 12, and *M* = 2 for Matataki, and default parameters were used for the other methods. The results (Additional file 1: Figure S3, Fig. 1) indicated that our method had the second best performance with respect to correlation (Additional file 1: Figure S3A, C, E, G and I; Fig. 1a except MatatakiSubset) and the minimum absolute mean difference among alignment free methods (Additional file 1: Figure S3B, D, F, H and J; Fig. 1b except MatatakiSubset). Because RSEM had the best performance for both correlation and error, using the result from this alignment-based method would be the best choice to evaluate prediction performance if the calculation costs are acceptable. In this analysis, we used all genes; however, some genes did not have any indexed k-mers, which cannot be managed by our method. Therefore, as a practical reference, we have provided the results obtained when excluding the genes without any indexed k-mers in Fig. 1a and b as the MatatakiSubset. Since we used RSEM’s RNA-Seq simulator for this evaluation, comparison with RSEM was not appropriate. Therefore, we used eXpress to compare the results with real data, which emerged as the best performance tool aside from RSEM and our method.

### Comparison of quantification quality using real data

#### Comparison of TPM

**Fig. 2:**
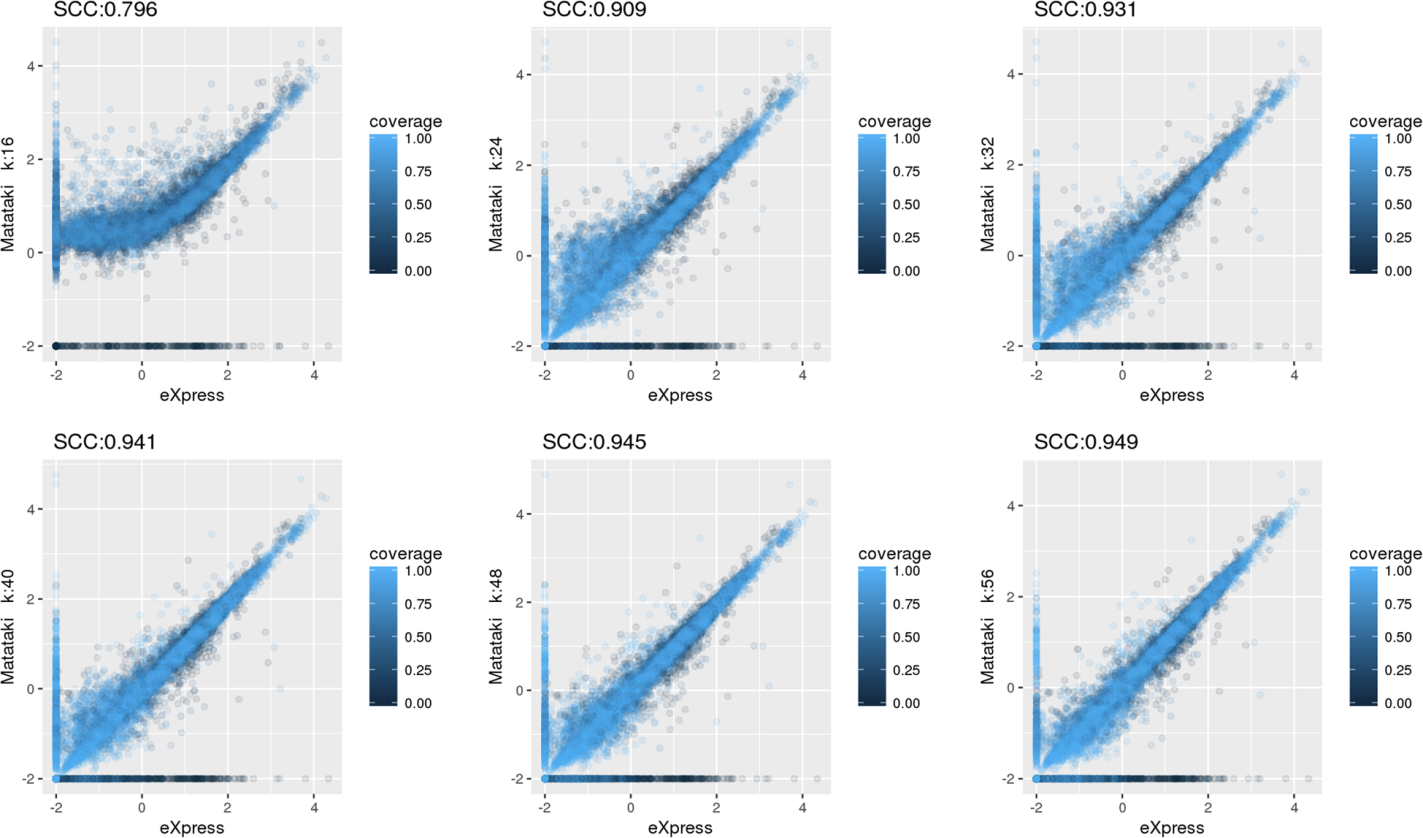
Comparison of TPM when *k* was varied. The x-axis shows the TPM values of eXpress, the y-axis shows the TPM values of our method, and the color indicates the indexed k-mer coverage of each gene when changing *k* from 16 to 56 with a step of 8

Figure 2 shows the comparisons of TPM values obtained with our method and eXpress for different *k* values. Our method gave similar TPM values for all *k* values, and larger *k* values provided better Spearman correlation coefficient (SCC) values, reaching up to 0.949 with *k* = 56*.* These results indicated that higher *k* values are preferable for better estimation; however, a large *k* is not always the best choice for a given analysis. For example, in the Short Read Archive, 9.2% of human RNA-Seq data have reads with a length shorter than 50. Accordingly, to cover 99% of human RNA-Seq data, *k* should be smaller than 34. Therefore, we used *k* = 32 in the following analyses, for which the SCC of TPM values obtained between our method and eXpress was 0.931. We summed the TPM values of a given gene for comparison with Matataki’s TPM.

We also determined the effect of the correlation of TPM values between eXpress and Matataki when changing the step size parameter *S* from 1 to 16 with a step of 4 (see Additional file 1: Figure S4). Overall, larger *S* values produced better correlations based on SCC values, suggesting that introducing the step size parameter *S* can reduce accidental matches of indexed k-mers with short reads. Usually, an indexed k-mer is matched in a successive way and forms a few cover islands, whereas accidental matches will show a different pattern and can therefore be eliminated by skipping all matches. Similar to the considerations for selecting *k* values, an *S* value that is too large will be problematic; therefore, we used *S* = 12 for the following analyses as a representative value showing a sufficient degree of correlation with the existing method.

We further checked the effects of the accept-count *M* parameter by changing it from 1 to 4 (Additional file 1: Figure S5). This parameter was introduced with the aim of avoiding the mis-assignment of some reads to genes due to accidental matches between indexed k-mers and the reads. We found that the SCC value was better with *M* > 1 than with *M* = 1, indicating that some reads were actually counted as mis-assigned genes. However, the SCC value was worse at *M* = 4 than at *M* = 3. These results indicated that a certain level of mis-assignment should be allowed for more accurate quantification.

The mapping rate is also an important measure for evaluating the performance of the method. We compared mapping rates by varying *k*, *S,* and *M*. As expected, the mapping rate became smaller as *k* became larger, because the matching condition was stricter for larger *k* values (see Additional file 1: Figure S6A). When *k* = 16, the mapping rate exceeded the rate of bowtie, indicating that *k* = 16 may be too small to avoid accidental matches of indexed k-mers and the resulting mis-assignment of the read to genes. In a similar way, larger *M* values resulted in lower mapping rates, as expected (Additional file 1: Figure S6C). In particular, the mapping rate dropped rapidly at *M* = 4, suggesting that *M* = 4 may be too strict for these data. By contrast, *S* only had a minimal effect on the mapping rates (Additional file 1: Figure S6B), and selection of the *S* parameter was not problematic in this case. Thus, selection of the best combination of *k*, step size *S,* and accept-count *M* is one of the problems that must be addressed in implementing the method, which will depend on the read length and experimental qualities.

When *k* = 32, the number of genes without indexed k-mers was 717. The details of these uncovered genes are shown in Table 1, and the full coverage list of transcripts is shown in Additional file 3: Table S1. Half of the uncovered genes were non-coding genes. Because non-coding genes cannot be amplified in the translation step, a high copy number in the genome is required for functional activity. The other half of the uncovered genes were protein-coding genes. Noted that paralogous genes can be one of the causes of finding non-unique k-mers. According to the HomoloGene group, but only 21.1% of paralogous genes were uncovered. (see Additional file 3: Table S2). We also performed enrichment analysis of the uncovered genes with TargetMine [24], which revealed five biological-process Gene Ontology (GO) terms (Additional file 3: Table S3) and four molecular function GO terms (Additional file 3: Table S4) that were significantly enriched. Since genes related to ubiquitin and defense response have many paralogous genes, these GO terms were particularly enriched.

**Table 1:** Details of the uncovered genes

Type of Gene	Number of uncovered genes	Total number of genes	Percentage of uncovered genes
Non-coding RNA	393	6250	6.3%
MicroRNA	233	1880	11.9%
Ribosomal RNA	19	21	90.5%
Small nuclear RNA	35	109	32.1%
Small nucleolar RNA	45	390	11.5%
Other non-coding RNA	61	3850	1.6%
Pseudo-gene	21	927	2.7%
Protein-coding gene	303	18,720	1.6%
Paralogous gene	137	505	27.1%

### Comparison of CPU time and memory usage

**Fig. 3:**
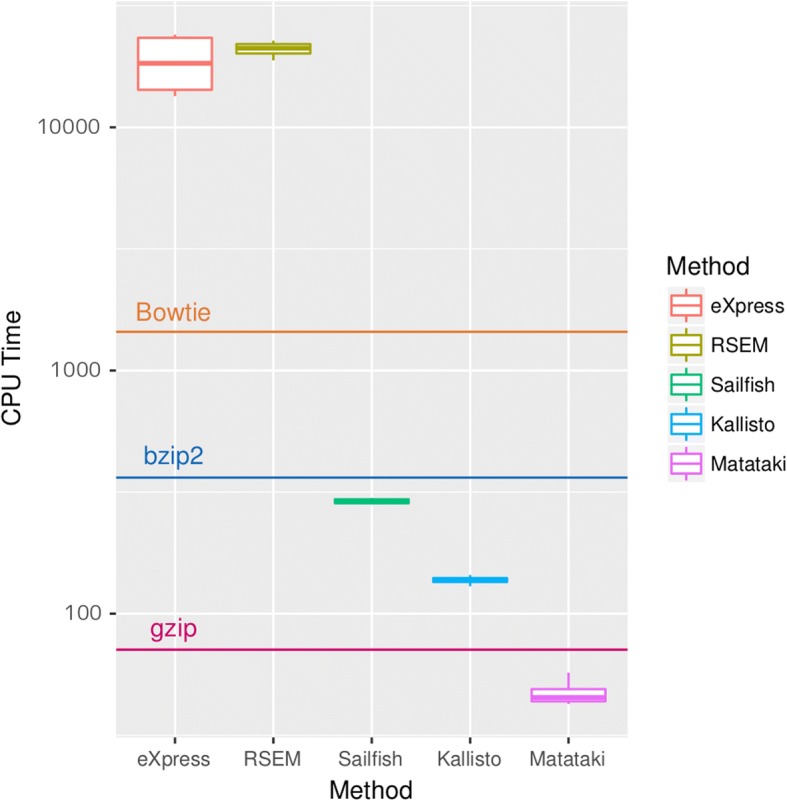
Comparison of CPU time for different methods

We compared the CPU time and memory usage of six existing methods with those of Matataki using real data in four runs, ERR188074, ERR188125, ERR188171, and ERR188362. In this comparison, we used *k* = 32, *S* = 12, and *M* = 3 as the parameters. The results confirmed that our method was much faster than the alignment-based methods bowtie without quantification, RSEM, and eXpress. Matataki was twice as fast as the alignment-free methods Sailfish and Kallisto (Table 2, Fig. 3). With respect to memory usage, Matataki used 3.5 GB RAM, while the other methods used 3.8 GB or more RAM. It should be noted that Matataki was also faster than gzip (~ 55 s) and bzip2 (~ 285 s).

**Table 2:** Comparison of running times among methods

Run accession		ERR188074	ERR188125	ERR188171	ERR188362
Run and mapping statics	Number of reads	31,540,813	28,810,860	30,386,179	26,255,381
	Length of reads	75	75	75	75
	Bowtie mapping rate	84.7%	80.2%	84.6%	80.4%
CPU time comparison (s)^a^	eXpress	14,546.6	24,036.1	13,429.5	23,103.9
	RSEM	22,700.6	20,545.9	21,753.1	18,842.2
	Bowtie	1487.8	1477.5	1472.6	1319.5
	Sailfish	299.0	281.0	294.2	285.5
	Kallisto	138.7	144.2	136.7	129.5
	Matataki	57.2	46.4	43.9	42.5
Acceleration rate compared with existing methods	eXpress	254	517	305	543
	RSEM	397	442	495	443
	Bowtie	26.0	31.8	33.5	31.0
	Sailfish	5.23	6.05	6.69	6.71
	Kallisto	2.43	3.107	3.11	3.05

^a^Values represent the median for 10 measurements

It should be noted that our approach is not designed for precise quantification of transcripts and minor expressed genes. The speed of quantification takes priority over these limitations in our method because increasing the amount of RNA-Seq data improves the value of reanalysis, such as the quality of gene co-expression network [17].

### Expected use-cases and limitations

Since Matataki was designed with the objective of improving the speed of quantifying RNA-Seq data, the accuracy of quantification can be worse than that of other methods. Therefore, Matataki is suitable for large-scale reanalysis such as searching similar gene expression profiles or gene co-expression. As shown in Additional file 1: Figure S7, a larger number of samples in gene co-expression improves the accuracy of GO term prediction. Since the amount of RNA-Seq data is rapidly increasing in public databases, it is important to increase the number of reanalyzed samples to determine gene co-expression patterns.

Nevertheless, Matataki is not suitable for common RNA-Seq purposes because other methods are sufficiently fast and provide better accuracy. For example, a single nucleotide substitution has larger effects in Matataki than in other methods, because even a single point substitution changes the k-mer for 2 *k* − 1 bases, which ultimately affects the number of k-mers in a transcript and calculation of the TPM value. It was also previously reported that transcript-level abundance inference improves gene-level expression estimation, both theoretically [25] and practically [18]. Another weak point of this method is that the ratio of uncovered genes was over half when we used GENCODE version 28 [21] to create the index, because the comprehensive GENCODE annotation includes many incomplete transcripts without a start codon and stop codon (see Additional file 3: Table S5). Since Matataki requires unique k-mers between genes and common k-mers among transcripts, major transcripts should be selected as reference transcripts. For these reasons, the expected use-case of Matataki is in the large-scale reanalysis of RNA-Seq data, such as for gene co-expression or searching similar expression profiles.

## Conclusion

We present Matataki, a much faster and user-friendly quantification method for RNA-Seq data analysis. This method archived the data at a rate more than 300 times faster than achieved with the alignment-based method bowtie/eXpress and two times faster than that achieved with other alignment-free methods, and had smaller memory requirements. In addition, Matataki had shorter calculation times, comparable quantification accuracy levels to alignment-based methods, and better accuracy than alignment-free methods. Because Matataki was even faster than decompressing gzip and bzip2, the improved computational cost and speed of Matataki resolves one of the major limitations of RNA-Seq analyses, shifting the bottleneck to decompression from mapping reads.

## Additional files

Additional file 1: Supplementary methods (pseudocode and mapping) and figures. (DOCX 1581 kb)

Additional file 2: Source code of Matataki. (GZ 7760 kb)

Additional file 3: **Table S1.** Numbers of indexed k-mer for each transcript. **Table S2.** List of paralogous genes and number of indexed k-mers. **Table S3.** List of enriched biological process GO terms in uncovered genes. **Table S4.** List of enriched molecular function GO terms in uncovered genes. Table S5: Details of the uncovered genes in GENCODE transcripts. (XLSX 3579 kb)

## Abbreviations

EM: Expectation maximization
FPKM: Fragment per kilobase million
SCC: Spearman’s correlation coefficient
TPM: Transcripts per million
