# Supplementary material for: Matataki: an ultrafast mRNA quantification method for large-scale reanalysis of RNA-Seq data
Source: BMC Bioinformatics. 2018 Jul 16;19:266. doi: 10.1186/s12859-018-2279-y (PMC6048772; doi:10.1186/s12859-018-2279-y)
Supplement: Supplementary file 1 — Supplementary methods (pseudocode and mapping) and figures. (DOCX 1581 kb) [file 12859_2018_2279_MOESM1_ESM.docx]

Additional File 1 for

Matataki: An ultrafast mRNA quantification method for large-scale reanalysis of RNA-Seq data

**Method S1: Create the index pseudocode**

for gene in *a gene list*:

for transcript in *transcripts of the gene*:

for k-mer in *clipped k-mers from the transcript*:

if k-mer *was found in other genes*:

continue

if k-mer *was not found commonly in other transcripts of the gene*:

continue

*add* k-mer *to index*

*add reverse complement of* k-mer *to index*

*count the number of k-mers in* gene

**Method S2: Mapping the pseudocode**

for read in *a FASTQ file*:

i = 0

m = 0

while i + *k* < *length of* read:

k-mer = *substring of the read from* i *with length* *k*

if the k-mer was found in the index:

if *an associated gene is different from previously reported*:

*classify the read to unmapped*

break

m += 1

if m >= M:

*assign the read to the associated gene*

break

i += *S*

**Figure S1**. (A) Coverage of base pairs with indexed k-mers. (B) Coverage of genes with one or more indexed k-mers

**Figure S2**. (A) Nucleotide coverage by gene. (B) Number of cover islands. (C) Lengths of the longest and second longest cover islands for each gene.

**
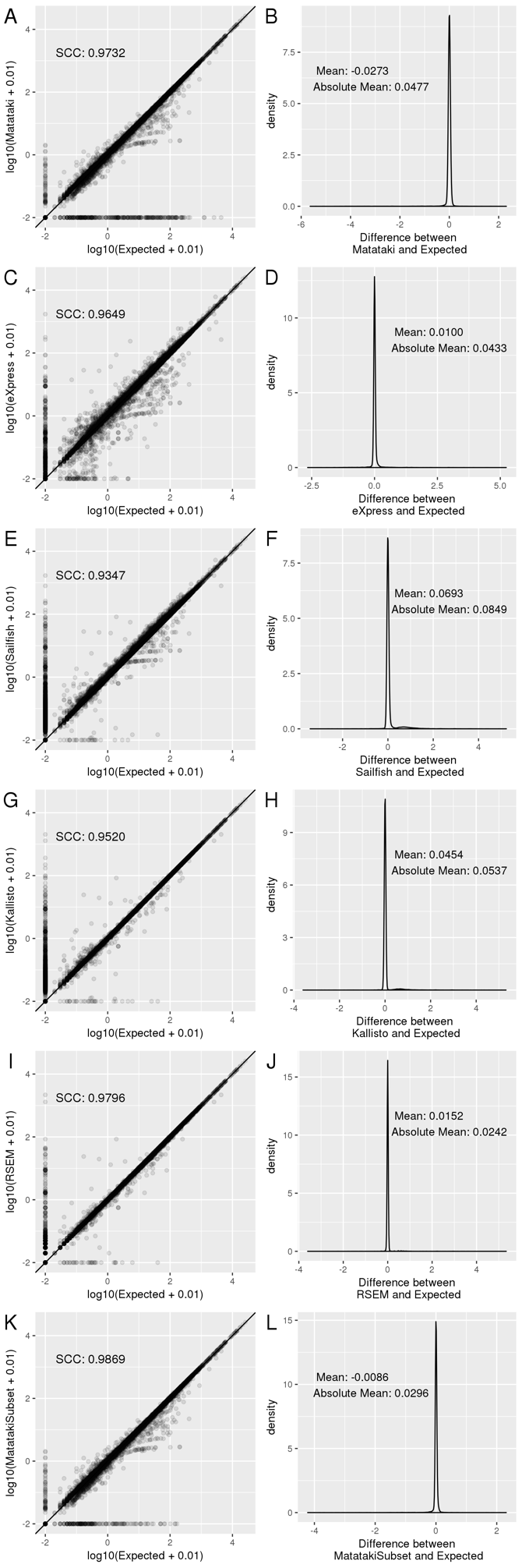
Figure S3.** Comparison of transcript per million (TPM) values between the expected results and estimated results using simulation data. **(A)** Scatter plot of expected and estimated gene expression levels using the proposed method. SCC, Spearman correlation coefficient. **(B)** Density plot of the differences between expected and estimated gene expression levels using the proposed method. **(C, D)** Scatter plot and density plot using eXpress. **(E, F)** Scatter plot and a density plot using Sailfish. **(G, H)** Scatter plot and a density plot using Kallisto. **(I, J)** Scatter plot and a density plot using RSEM. Since we used RSEM to generate the simulated RNA-Seq data, RSEM showed the best performance. **(K, L)** Scatter plot and density plot using the proposed method. Genes without indexed k-mers were excluded from these figures.


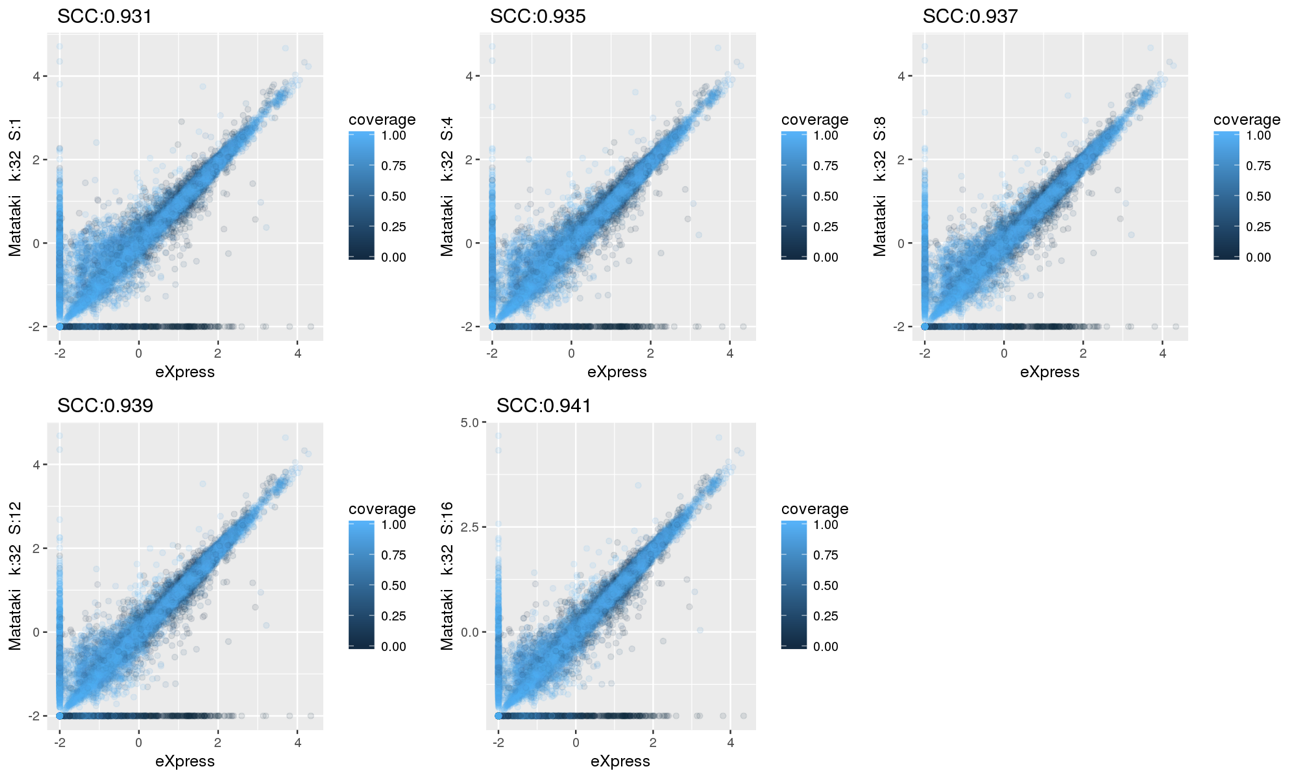


**Figure S4.** Comparison of transcript per million (TPM) values between Matataki and eXpress when varying the step size parameter *S* keeping *k* constant at 32. SCC, Spearman correlation coefficient.


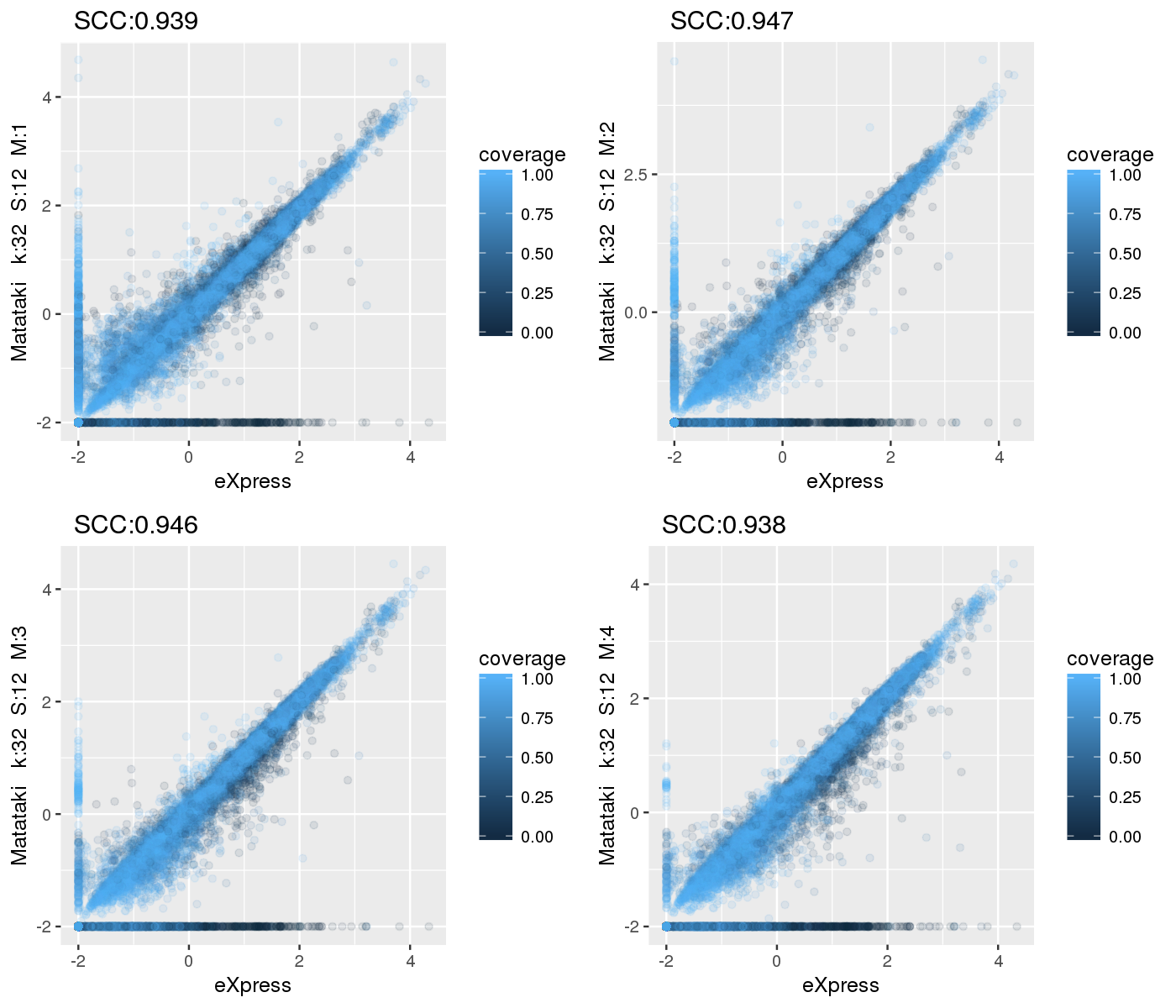


**Figure S5.** Comparison of transcript per million (TPM) values between Matataki and eXpress when the accept-count parameter *M* was varied, keeping the step size *S* constant at 12 and the *k* constant at 32. SCC, Spearman correlation coefficient.

**Figure S6.** Effect of varying parameters on the mapping rates.

**Figure S7. Performance of GO term prediction when the number of samples was varied.**

Large-scale gene co-expression analysis is a powerful approach to inspect relationships among genes. We applied Matataki to calculate the gene co-expression database COXPRESdb [1], and evaluated the performance of obtaining co-expression data as described in Ref. [2] while varying the number of samples. Since gene co-expression analysis is used for predicting protein complexes or pathways, we focused on the high-sensitivity region in the evaluation. Therefore, we used the partial area under curve (pAUC) with sensitivity higher than 0.95 to evaluate the performance of gene co-expression. We found that the number of samples was highly correlated with the performance of gene co-expression analysis.

[1] Okamura Y, Aoki Y, Obayashi T, Tadaka S, Ito S, Narise T, et al. COXPRESdb in 2015: coexpression database for animal species by DNA-microarray and RNAseq-based expression data with multiple quality assessment systems. Nucleic Acids Res. 2015;43:D82–6; <https://doi.org/10.1093/nar/gku1163>

[2] Obayashi T, Kinoshita K. Rank of correlation coefficient as a comparable measure for biological significance of gene coexpression. DNA Res. 2009;16:249–60; <https://doi.org/10.1093/dnares/dsp016>
